# Psychological distress, perceived stress, and public stigma in skin neglected tropical diseases in Ghana: A structural equation model of a cross-sectional survey

**DOI:** 10.1371/journal.pntd.0013387

**Published:** 2025-07-31

**Authors:** Samuel Adjorlolo, Stephanopoulos Kofi Junior Osei, Emma Efua Adimado, Mawuko Setordzi, Vincent Valentine Akorli, Lawrencia Obenewaa Aprekua, Paul Adjorlolo

**Affiliations:** 1 Department of Mental Health, School of Nursing and Midwifery, University of Ghana, Accra, Ghana; 2 Research and Grant Institute of Ghana, Accra, Ghana; 3 Department of Epidemiology, School of Public Health, University of Ghana, Accra, Ghana; 4 Department of Research, Education and Administration, School of Nursing and Midwifery, University of Ghana, Accra, Ghana; 5 School of Nursing and Midwifery, University of Health and Allied Sciences, Ho, Ghana; 6 Department of Psychology, School of Social Sciences, University of Ghana, Accra, Ghana; 7 Department of Biostatistics, School of Public Health, University of Ghana, Accra, Ghana; Keele University, UNITED KINGDOM OF GREAT BRITAIN AND NORTHERN IRELAND

## Abstract

**Background:**

Individuals affected by skin Neglected Tropical Diseases (skin-NTDs) are at increased risk of psychological distress, public stigma and perceived stress. However, the nexus between these burdens remains underexplored globally. This study investigates the burden of psychological distress and perceived stress, their relationships with public stigma and the moderating effects of sociodemographic factors on these relationships among individuals affected by skin-NTDs in Ghana.

**Methods:**

A cross-sectional study design was utilized. Data were gathered from 292 conveniently sampled individuals with skin-NTDs in the Nkwanta North and South Districts of the Oti region in Ghana. A structured questionnaire that assessed perceived stress, psychological distress and public stigma was administered. The prevalence of psychological distress and perceived stress was estimated using descriptive statistics, while Structural Equational Modelling (SEM) was used to test the hypothesised relation among the study variables and moderating effects of sociodemographic variables.

**Results:**

Approximately 42.8% of the participants experienced psychological distress, and 61.1% reported elevated levels of perceived stress. SEM revealed that public stigma was significantly associated with psychological distress (β = 0.26, p < 0.001, SE = .05) and perceived stress (β = 0.52, p < 0.001, SE = .04). Perceived stress significantly mediated the relationship between public stigma and psychological distress (β = 0.26, p < 0.001) The relationship between public stigma and perceived stress was moderated by educational level (p = 0.03) and help-seeking behavior for mental health (p < 0.05).

**Conclusion:**

The study highlights the complex relationship between public stigma, perceived stress and psychological distress among individuals affected by skin-NTDs, highlighting the need for targeted strategies to mitigate their impacts..

## Author summary

Individuals affected by skin-NTDs experience negative behaviours that significantly threaten their quality of life. Among them are (1) psychological distress, referred to as a combination of anxiety and depressive symptoms, (2) public stigma, and (3) perceived stress. The burden imposed by these negative experiences are enormous, necessitating the need for empirical studies to characterize the nature of their relationships and the sociodemographic factors that modulate these relationships. This interesting line of research has been barely conducted in the skin-NTDs literature, globally, leaving a conspicuous gap in our understanding. To this end, we recruited 292 participants affected by skin-NTDs in local communities in Ghana who responded to measures of psychological distress, public stigma, and perceived stress. The results of data analyses showed that more than 50% of the participants reported significant perceived stress and psychological distress. Second, an increase in exposure to a burden (e.g., public stigma) leads to a concomitant increase in experience of other burden (e.g., psychological distress). Furthermore, it was observed that the effect of public stigma on the experience of psychological distress was mediated by perceived stress. Lastly, participants with high education and those who have sought help for mental health in the past were less likely to experience significant perceived stress following exposure to public stigma. The outcome of the study has the potential to inform efforts to improve the psychosocial wellbeing and quality of individuals affected by skin-NTDs as well as contribute to the overall goal of eradicating skin-NTDs in Ghana and other jurisdictions.

## Introduction

In Sub-Saharan Africa (SSA), Neglected Tropical Diseases (NTDs) continue to pose a significant public health challenge [1]. Despite the substantial decline in NTD cases (over 90% reduction in the past four decades), SSA still has the highest age-standardised prevalence rate and incidence of NTDs [2].

Like many other SSA contexts, Ghana bears a considerable burden of skin-NTDs, despite witnessing some remarkable and positive progress in tackling NTDs. A historical review of Ghana’s NTD trajectory has revealed a significant decrease in the prevalence of some NTDs from 2000 to 2017, including schistosomiasis (-32%), leprosy (-26%), guinea worm (-100%) and lymphatic filariasis (-92%). In contrast, others such as hookworm infestation (+9%) and dengue (+338%) witnessed an increase [3]. Of interest are skin-NTDs which constitute a sub-type of NTDs that manifest explicitly on the skin or visible areas of the body. Notable among them are buruli ulcer, leprosy, cutaneous leishmaniasis and lymphatic filariasis. The skin lesions in cutaneous leishmaniasis are usually found on exposed areas, especially the face, arms and legs and can be troublesome and unsightly [4]. Healed cutaneous leishmaniasis leaves a scar, often referred to as “beauty scar” [5]. A community-based cross-sectional study reported 31.9% prevalence rate of cutaneous leishmaniasis in Nkwanta North and South Districts in Ghana [6].

With limited awareness and access to safe water and sanitation, the country’s vulnerable and impoverished communities continue to be at a significant risk of skin-NTDs [6]. As preventive measures have been intensified over the past decades to end skin-NTDs in Ghana [7], it is crucial to improve the quality of life of all individuals affected by these conditions [8]. These individuals often experience mental health challenges, including psychological distress, operationalized as the combined symptoms of depression and anxiety, that threatens their overall wellbeing [8–10]. A recent systematic review reported that 7% to 54% of individuals with buruli ulcer, lymphatic filariasis and leprosy living in low- and middle-income countries (LMICs) experienced various degrees of psychological distress [10]. The prevalence of psychological distress among individuals with leprosy from different countries reportedly ranged from 13% to 71% [11–13]. Psychological distress among individuals with skin-NTDs stem from a complex interplay of several factors, including pain, discomfort and physical disability [7,14]. In addition to these health condition-related challenges, social and demographic factors, particularly stigma, discrimination, social isolation, low education, poverty/low economic status, female gender, functional impairment, partner neglect and violence, and relationship difficulties have been implicated in the increasing burden of psychological distress in skin-NTDs [9,10,15–17].

The diagnosis of skin-NTDs tend to invoke and potentiate stressful moments which include repeated visits to healthcare centers for a series of medical tests and assessments. Commuting between units/departments within and/or between hospitals for healthcare is a common feature of the Ghanaian healthcare system which can add to the stress level of individuals affected by skin-NTDs. The poor road infrastructure between communities and healthcare institutions and the deplorable nature of the transport system in impoverished communities in Ghana are major sources of stress experiences among individuals seeking healthcare. Finally, the restrictions imposed by the management of skin-NTDs in the form of new behavioural practices such as taking medications can be interpreted as stressful. These stressful experiences can culminate into psychological distress.

Closely related to the above is the experience of stigma, which mainly stems from the cultural and spiritual meanings associated with skin-NTDs conditions as well as their disfigurement sequelae [18]. Goffman describes stigma as a response to characteristics that are perceived as dishonourable or shameful [19]. Stigma can manifest in various forms [20], including public stigma, which refers to as a set of negative attitudes and beliefs that precipitate rejection, discrimination and fear of people with “unpleasant look and behaviors” (in this case skin-NTDs) [21]. Compared with other forms of stigma, public stigma has been implicated in poor engagement with mental health and social services, segregation and negative treatment outcomes [22]. Public stigma can contribute to the perceived stress experience and psychological distress among individuals affected by skin-NTDs.

### The present study

Although the intersection between psychological distress, perceived stress and public stigma among individuals affected by skin-NTDs is evident [10], Ghana’s efforts to address skin-NTDs have been predominantly focused on biomedical approaches, such as disease surveillance, mass drug administration, and case management [23,24]. This has also been the case with the country’s skin-NTD research efforts which have been characterized by sparse investigations into the stigma experiences and psychological distress of individuals affected by skin-NTDs [10, 25]. As such, there is limited understanding of how public stigma interacts with psychological distress and perceived stress burden in the Ghanaian context and the broader sub-Saharan African (SSA) region, among others. Insight into the interaction and influence of these factors in respect of skin-NTDs will not only contribute to the burgeoning literature but, more importantly, it will help to inform psychosocial-based intervention programs to improve the mental health and wellbeing of this vulnerable population. It would also guide efforts to integrate mental health into primary and community care of individuals affected by skin-NTD by facilitating the screening and provision of psychosocial interventions, including referral services. This is envisaged to boast ongoing efforts to decentralize and integrate mental health services into primary and community healthcare system in Ghana. Consequently, this study investigates the burden of psychological distress and perceived stress, their relationships with public stigma, as well as the moderating effects of sociodemographic factors on these relationships, using data collected from individuals affected by skin-NTDs in Ghana.

**The study objectives are:**

1. Assess the prevalence of psychological distress and perceived stress among individuals affected by skin-NTDs.
2. Determine the relationships among psychological distress, perceived stress, and public stigma in individuals with skin-NTDs.
3. Identify potential moderating factors that influence the relationships between psychological distress, perceived stress, and public stigma among individuals affected by skin-NTDs.

### Study hypotheses

- H_1_: There is a positive association between public stigma, perceived stress and psychological distress
- H_2_: Perceived stress would significantly mediate the relationship between public stigma and psychological distress.
- H_3_: Sociodemographic variables (e.g., educational background and mental health help-seeking) will significantly moderate the relationship between public stigma, perceived stress and psychological distress.

## Method

### Ethics statement

This study received ethics approval from Ghana Health Service Ethics Review Committee (GHS-ERC: 023/03/23). Written informed consent was obtained from interested participants by signing or thumbprinting the consent form. This was done after the participants demonstrated (e.g., present a summary of the research) understanding of the study, including their responsibilities. Principles of research ethics including confidentiality, anonymity and protection from harm were assured and upheld.

### Study setting

The study was carried out in Nkwanta North and South Districts in the Oti Region of Ghana. These two districts were purposively selected because communities in these districts are known to be co-endemic for skin-NTDs such as Buruli ulcer and onchocerciasis. Most residents in these districts engage in farming and fishing as their main occupations and, as such, are always in close contact with infectious vectors, domestic animals, and livestock. Moreover, socio-economic inequalities and challenges are evident in the region, directly affecting access to healthcare services, proper housing, safe water, and sanitation. This situation creates an enabling environment for various disease categories, including skin-NTDs, to develop [6].

### Study design

The study utilized a cross-sectional design with an interviewer-administered questionnaire to capture the current situation in the Nkwanta districts [26]. Although cross-sectional designs do not provide a mechanism to monitor a particular behaviour over time, they help to understand behaviours to inform more robust research designs.

### Study participants and sample size

The study population consisted of individuals affected by skin-NTDs. To estimate the sample size required for this study, we used the prevalence rate of 7% (the lowest reported prevalence of depression and anxiety in individuals with skin-NTDs in a recent systematic review [10]. Assuming a 95%confidence level and a margin of error of 0.03, the minimum sample size was 278 participants. A total of 292 participants were eventually recruited. The sample include individuals aged 18 years and above, residents of Nkwanta North and South Districts in the Oti region of Ghana and diagnosed with skin-NTD at least 6 months prior to the commencement of the study. A convenience sampling method was employed to select participants based on their availability and willingness to take part in the study. The participants were recruited from their respective homes.

### Data collection measures

*Patient Health Questionnaire-4 (PHQ-4)* [27] was used to measure psychological distress (anxiety and depression), consistent with previous studies [28,29]. The PHQ-4 four items are rated on a four-point Likert scale, ranging from Not at all [0] to Nearly every day [4]. Sample items are [1] feeling down, depressed or hopeless and [2] feeling nervous, anxious or on edge. The tool is a reliable and valid screener for psychological distress, with a cut-off point of ≥3 yielding specificity and sensitivity of 94% and 77%, respectively [30]. A larger study by Apputhurai et al. [31] involving 54,127 participants from 24 countries, including SSA countries, reported that the PHQ-4 demonstrates a good convergent validity (Pearson’s correlation coefficients >±0.4) and good internal consistency (Cronbach’s α > 0.75). The PHQ-4 was also chosen for its brevity (i.e., 4-items), suggesting that it can be used to make quick impression about the psychological distress of individuals affected by skin-NTDs The Cronbach’s alpha reported in the current study was 0.97.

*Public stigma subscale of the 12-item HIV stigma scale version 12* [32] was adapted to assess public stigma in relations to skin-NTDs The public stigma sub-scale in particular had good internal consistency (Cronbach’s α = 0.78) and sufficient construct validity (all factor loading > 0.6) in previous studies [33]. The choice of the public stigma sub-scale was also influenced by the observation that items are culturally appropriate and friendly. The subscale contains four items, rated on a five-point Likert scale, ranging from strongly disagree [0] to strongly agree [4]. A sample item reads “People with wounds, sores or diseases on their skin are treated like outcasts”. Total scores were obtained by summing the individual scores, with higher scores reflecting more experiences of public stigma. A Cronbach alpha of 0.93 was reported in the current study.

*Perceived Stress Scale-4* (PSS-4) [34] was used to estimate the stress level of the participants. The PSS-4 is a 4-item scale rated on a 4-point Likert scale ranging from 0 (Never) to 4 (Very often). The tool has demonstrated good reliability and validity indices in earlier studies [35–37]. Items on the scale include being unable to control the important things in life and being confident in the ability to handle personal problems due to the presence of skin-NTDs. Higher scores indicate high perceived stress. The internal consistency (Cronbach’s alpha) was 0.93 in the current study.

*Background characteristics variables:* The study also collected data on the type of skin-NTDs which were initially self-reported by the study participants and confirmed from medical records. Demographic data captured included gender (male, female, or other), age (recorded in years as a continuous variable), marital status, household size, number of children, occupation, and education. Data on help-seeking behaviour for mental health (classified as prior medical consultation, traditional/professional mental health support, or no prior consultation) and water sources were categorized based on accessibility and quality, including piped water and surface water (such as rivers or lakes).

### Procedure for data collection

The survey was conducted using structured interviewer-administered questionnaires. The questionnaire, which comprised seven sections, including demographic information, mental health symptoms, experiences of stigma, overall quality of life, and access to mental health services, was part of a larger project aimed at elucidating strategies to integrate mental health into routine primary and community care. The questionnaire was meticulously designed and refined to ensure its relevance to the study’s objectives and settings. The structured questionnaire was translated by the Bureau of Ghanaian Languages, a government agency responsible for promoting local Ghanaian languages, into the common languages spoken in the study area (e.g., Twi and Likpakpani) and back-translated into English to check for accuracy and consistency. Field officers from Nkwanta South and North Districts who were familiar with the various communities where participants with skin-NTDs live were recruited and trained in sample selection and questionnaire administration, as well as community entry and exit. All the field officers completed standardized training sessions on how to administer the questionnaires in either Twi or Konkomba/Likpakpani. Regular supervision and monitoring of the data collection process were conducted to identify and resolve any issues. Using convenience sampling, individuals with skin-NTDs who were available and willing to participate in the study were approached to complete the questionnaire.

The participants were recruited from communities in Nkwanta North and South with high burden of skin-NTDs. The communities were selected with the support of the local healthcare officials (i.e., Disease Control Officers) working on skin-NTDs in the various Districts. The communities include Keri and Gekorang, Ashiabre and Dawa Kora, Agou Kunji of Nkwanta South District, as well as Obunja, Sibi central and Jato Kparikpari, and Sibi Hilltop in Nkwanta North. They were recruited in their respective homes with the support of the local healthcare officials who have established robust working relationship with individuals affected by skin-NTDs both in the community and hospital. The participants were given ample time to ask questions or express concerns that might affect their participation in the study. Questionnaires were administered to the participants, individually in their respective homes/communities and at locations that guaranteed less interruption and adequate to ensure privacy and confidentiality. For most participants, data were collected under a near-by tree used by the community members as a resting and gathering place. Data were collected in one session with each participant.. The data collection spanned June to July 2023.

### Data processing and analysis

Data analysis and processing were conducted using STATA 17 and SPSS AMOS 26. All variables were assessed for missing data and outliers. Continuous data were assessed for normality. Continuous variables that did not have a normal distribution were transformed to facilitate parametric analysis. Categorical variables, including education and marital status, were recoded to support moderation analysis. Summary statistics (mean and standard deviations) were computed for continuous variables, whereas frequencies and percentages of categorical outcomes (including prevalence of stress and psychological distress) and background characteristics were also reported.. A confirmatory factor analysis was conducted to evaluate the factor structure of the study measures. Model fit was assessed using standard goodness-of-fit indices, including Comparative Fit Index (CFI), Tucker-Lewis Index (TLI), Root Mean Square Error of Approximation (RMSEA), and Standardised Root Mean Square Residual (SRMR), following fitting guidelines [38]. The hypothesised relationship among psychological distress, perceived stress and public stigma was tested using Structural Equation Modelling (SEM). The indirect effect of public stigma on psychological distress through perceived stress was assessed using bootstrapping to calculate a bias-corrected confidence interval. Moderation analyses were conducted to identify key factors that influence the relationships in the model. The SEM model was assessed using the goodness of fit indices mentioned earlier.

## Results

### Background characteristics of study participants

Out of a total of 300 participants who were invited to participate in the study, 292 completed the survey, representing a 93% response rate. Table 1 presents the background characteristics of the study participants, with an average age of 38.9 years. About 56.9% of the participants were males, mostly farmers (83.7%) and living with one skin-NTD condition (93.7%). The majority were living with onchocerciasis and related ocular complications (n = 76, 26%), followed by cutaneous leishmaniasis (n = 63, 21.66%) and scabies (n = 60, 20.6%).

**Table 1:** *Background characteristics of the study participants* (N = 292).

Characteristic	Frequency(n)	Percentage (%)
**Sex**		
Male	166	56.9
Female	126	43.1
**Age (M = 38.9, SD = 15.9)**		
18-25 years	72	22.7
26-35 years	72	24.7
34 – 48 years	74	25.3
49-86 years	74	23.4
**Occupation**		
Farming	244	83.7
Fishing	6	2.
Trading	5	1.7
Others (unemployed etc.)	37	16.7
**Marital status**		
Married	208	71.2
Widowed	16	5.5
Single	64	21.9
Others	4	1.4
**Educational status**		
No formal education	188	64.4
Primary	57	19.5
Junior High School	35	12.0
Senior high school	11	3.8
Tertiary	1	0.3
**Number of children**		
None	66	22.6
One to three children	105	36.0
Four to seven children	101	34.6
More than seven children	20	6.9
**Family History of NTD**		
Yes	247	84.6
No	45	15.4
**Name of NTD**		
Buruli ulcer	36	12.3
Elephantiasis	6	2.1
Hydrocele	18	6.2
Leishmaniasis	63	21.6
Onchocerciasis	76	26.0
Ringworm	5	1.7
Scabies	60	20.7
Yaws	28	9.5
**Number of NTDs**		
One	284	97.3
Two	8	2.7
**Source of drinking water**		
River	95	32.5
Stream	114	39.0
Pipe	81	27.7
Other	2	0.7
**Seeking mental health related help from health professionals**		
Yes	172	58.9
No	120	41.1
**Seeking mental health related help from spiritualists/herbal list**		
Yes	171	58.6
No	121	41.4

### Prevalence of psychological distress among study participants

**Fig 1:**
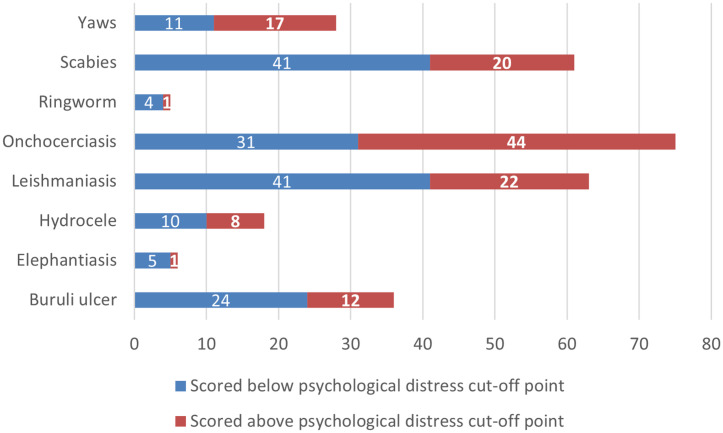
Distribution of psychological distress status by skin-NTD type.

The overall mean score of the participants on the psychological distress scale (PHQ-4) was 2.7 (SD = 1.1). About 42.8% (n = 125) obtained a score greater than or equal to the threshold for psychological distress (≥3). Table 2 presents a summary of the item-specific responses of participants. Among those with Onchocerciasis, 55.8% scored above the cutoff point for psychological distress (Fig 1). This was followed by participants with Yaws (57.1%) and Hydrocele (44.4%). In contrast, less psychological distress was observed among participants affected by Ringworm (20.0%), Elephantiasis (16.7%) and Cutaneous Leishmaniasis (15.9%).

**Table 2:** Participant reports of anxiety and depression symptoms by frequency in the past 2 Weeks.

Scale item	Not at all (%)	Several days (%)	More than half the days (%)	Nearly Every day (%)
Having Little interest or pleasure in doing things	16.8	40.0	11.3	34.9
Feeling down, depressed, or hopeless	14.7	38.0	12.3	34.9
Feeling nervous or anxious	14.4	38.0	12.7	34.9
Unable to stop or control worrying	13.36	36.3	15.4	34.9

### Prevalence of perceived stress among study participants

The participants’ overall mean perceived stress score was 2.9 (SD = 1.0). Approximately 61.1% (218) scored at or above the cutoff point (≥3) for perceived stress. The responses on the various perceived stress items are summarised in Table 3.

**Table 3:** Participant reports of stress in the past one month.

Scale item	Never (%)	Sometimes (%)	Often (%)	Very often (%)
Unable to control important thing in life	9.6	38.0	14.4	38.0
Lack of confidence in handling personal problems	9.3	34.6	16.6	39.7
Feeling things do not go as planned	8.2	29.5	19.2	43.2
Feeling difficulties piling up to the of them being overwhelming	10.6	27.7	19.9	41.8

**Fig 2:**
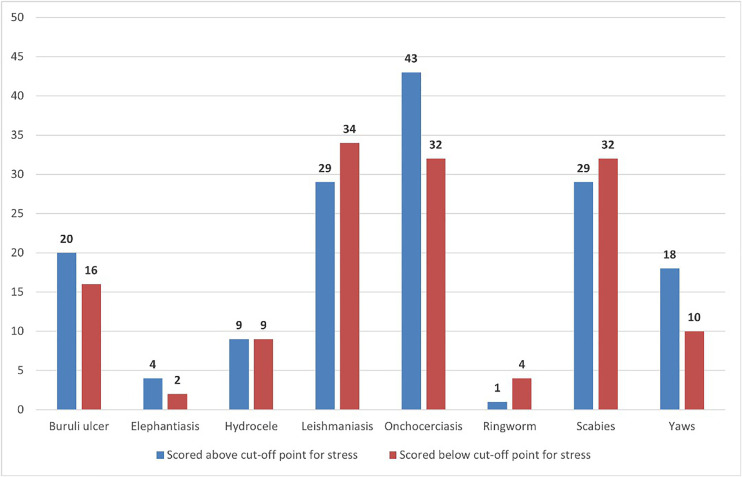
Distribution of stress by skin-NTD types.

Among participants who scored above the cutoff point (≥3) on the perceived stress scale, the highest proportion was observed among those affected by Yaws (60.7%), followed by Onchocerciasis (53.3%) and Elephantiasis (50.0%). Participants who reported lower perceived stress were those affected by Scabies (29.5%), Ringworm (20.0%), and Cutaneous Leishmaniasis (15.9%). These findings highlight the varying perceived stress burden associated with different skin-NTDs (Fig 2).

### Relationship between psychological distress, perceived stress and public stigma

**Fig 3:**
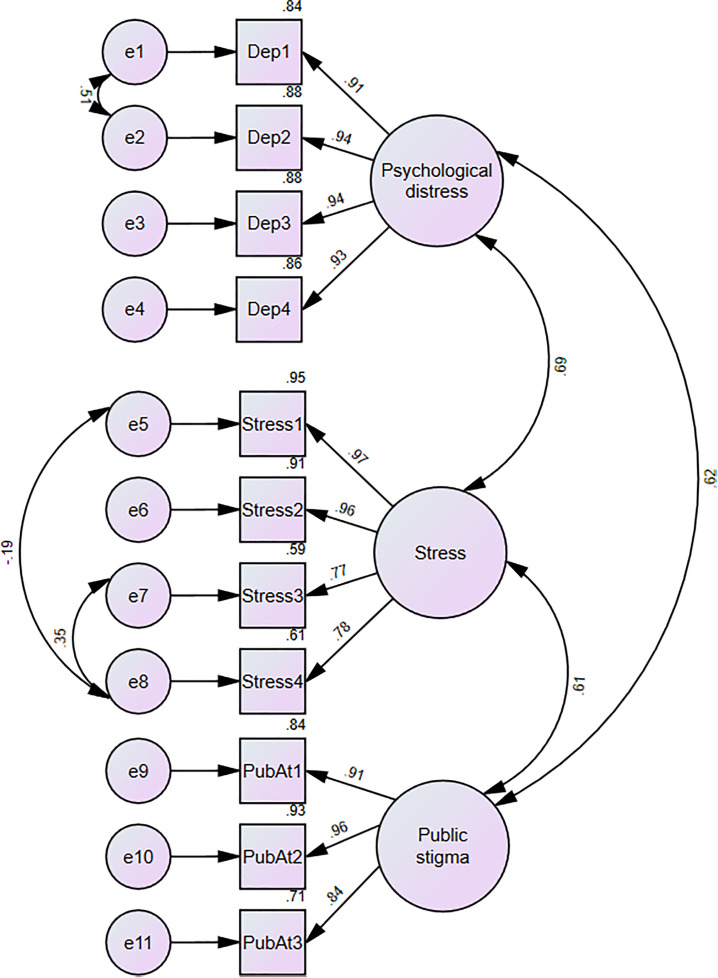
CFA model of depression, anxiety, perceived stress, and public stigma.

Prior to conducting SEM, CFA was used to assess the measurement model, where psychological distress, perceived stress and public stigma were latent variables with corresponding manifest variables (scale items). The CFA was performed using maximum likelihood estimation (Fig 3). The hypothesised model demonstrated an acceptable fit to the data based on several model fit indices after model specification in which the error variances of some items were allowed to correlate freely. Although the chi-square test was significant (*X*^2^ (38) = 65.8, p = 003), the fit indices suggested adequate model fit: CFI = 0.99, RMSEA = 0.05 (90% CI = 0.03-0.07), and SRMR = 0.03. The factor loadings of each manifest variable were significant (p < 0.01) and ranged from 0.77 to 0.97.

**Fig 4:**
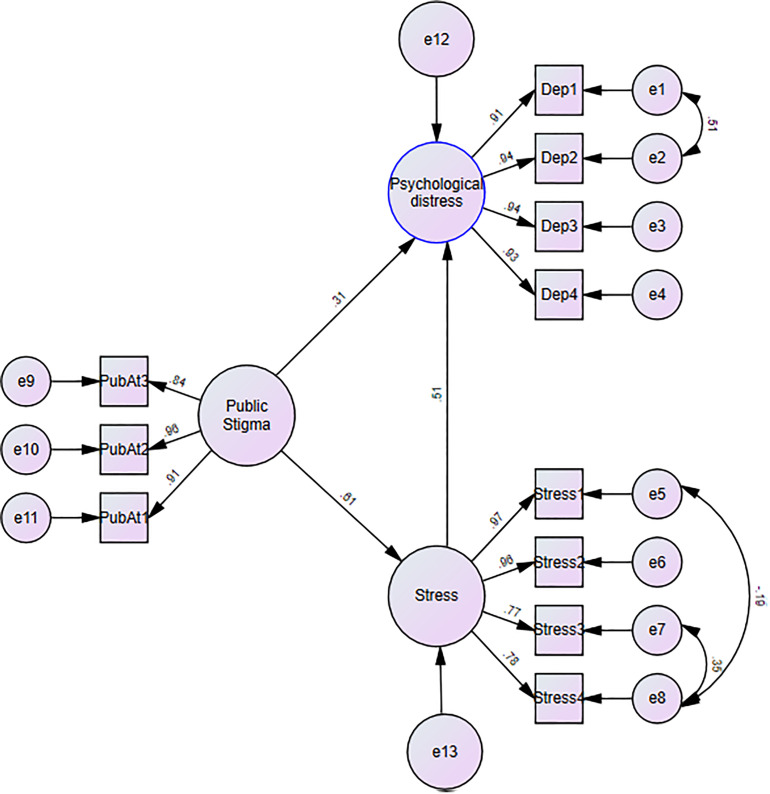
SEM depicting relationships among public stigma, perceived stress, and psychological distress.

SEM was conducted to assess the hypothesized relationships among psychological distress, perceived stress, and public stigma (Fig 4). The structural model demonstrated an acceptable fit to the data: χ² (38) = 65.8, p < 0.001, CFI = 0.99, TLI = 0.99, RMSEA = 0.05 (90% CI: 0.03–0.07), and SRMR = 0.05. All hypothesised paths were significant (p < 0.05). Specifically, public stigma was positively linked to psychological distress (β = 0.26, p < 0.001, SE = .05) and perceived stress (β = 0.52, p < 0.001, SE = .04). For every 1-unit increase in public stigma, psychological distress and perceived stress increased by 0.26 units and 0.52 units, respectively. Also, perceived stress was positively associated with psychological distress (β = 0.52, p < 0.001). The indirect effect of public stigma on psychological distress through perceived stress as a mediator was also significant (β = 0.26, p < 0.001).

### Moderation analysis of the relationship between psychological distress, stress and stigma

A multi-group SEM was conducted to assess the moderating effects of gender, marital status, education, mental help seeking from professionals and spiritualists/herbalists on the relationships between public stigma, perceived stress, and psychological distress. Tables 4 and 5 presents the model fit indices of the comparisons and moderation results. The path from public stigma to perceived stress was significantly stronger (*p* = 0.03) in individuals with no formal education (*β* = 0.60) compared to their counterparts with formal education (*β* = 0.39). The relationship between public stigma and perceived stress was also stronger (*p* = 0.03) in participants who sought mental help from health professionals (*β* = 0.54), compared to those who did not (*β* = 0.36). Seeking mental health from herbalist significantly moderated the relationship between public stigma and psychological distress (*p* = 0.03) and that of perceived stress and psychological distress (*p* = 0.01).

**Table 4:** Unconstrained model fit indices for each comparison in moderated SEM.

Moderator	χ² (df)	p-value	CFI	TLI	RMSEA (90% CI)
Gender	149.3 (76)	p < .001	0.98	0.97	0.06 (0.04–0.07)
Education	123.2 (76)	p < .001	0.99	0.98	0.05 (0.03–0.06)
Sought mental help from professionals	123.2 (76)	p < .001	0.98	0.98	0.05 (0.04–0.06)
Sought mental health (Herbalist)	131.9 (76)	p < .001	0.97	0.96	0.07 (0.05–0.08)
Marital status	164.6 (76)	p < .001	0.98	0.97	0.06 (0.05–0.08)

**Table 5:** Results of moderation effects of sociodemographic variables.

Moderator	Path	β (Group 1)	β (Group 2)	CMIN	DF	p-value
Gender	Stigma → Stress	**Males** 0.47	**Females** 0.59	1.72	1	0.19
	Stigma → Psychological distress	0.27	0.21	0.33	1	0.57
	Stress → Psychological distress	0.53	0.49	0.11	1	0.74
Formal Education	Stigma → Stress	**No** 0.60	**Yes** 0.39	4.54	1	0.03*
	Stigma → Psychological distress	0.25	0.32	0.40	1	0.53
	Stress → Psychological distress	0.49	0.51	0.03	1	0.87
Sought Mental Health (health professionals)	Stigma → Stress	**No** 0.33	**Yes** 0.50	3.6	1	0.06
	Stigma → Psychological distress	0.17	0.30	2.23	1	0.13
	Stress → Psychological distress	0.17	0.53	9.30	1	0.000*
Sought Mental Health (Herbalist)	Stigma → Stress	**No** 0.36	**Yes** 0.54	4.55	1	0.03*
	Stigma → Psychological distress	0.13	0.34	4.70	1	0.03*
	Stress → Psychological distress	0.27	0.57	6.31	1	0.01*
Marital status	Stigma → Stress	**No** 0.68	**Yes** 0.63	0.95	1	0.33
	Stigma → Psychological distress	0.47	0.49	0.71	1	0.40
	Stress → Psychological distress	0.54	0.52	0.01	1	0.92

## Discussion

This study assessed the burden of psychological distress, perceived stress, and public stigma among individuals affected by skin-NTDs. It also examined the relationships among these three factors and examined potential variables that may moderate these associations. Our findings revealed a high burden of psychological distress and perceived stress burden among individuals affected by skin-NTDs, aligning with previous systematic reviews on the psychosocial challenges faced by these individuals [10,39]. Although the current study cannot establish causality, the findings highlight that individuals living with skin-NTDs continue to suffer mental health challenges that could be linked to disfigurement, social rejection, discrimination, and the burden of care and recovery associated with skin-NTDs (5). This further emphasises the importance of integrating mental health support services into the management of skin-NTDs [40,41].

The role of public stigma in influencing psychological outcomes was one of the focal areas of this study. The SEM analysis demonstrated that public stigma was directly associated with higher levels of psychological distress and perceived stress burden. Participants who reported high levels of public stigma also tend to report high psychological distress and perceived stress burden, supporting the hypothesis that public stigma may exacerbate mental health issues in this population. Public stigma can manifest as social exclusion, internalised shame, reduced access to support systems, and livelihoods [42,43]. The interaction analysis also indicated that perceived stress mediated the relationship between public stigma and psychological distress. Public stigma has been reported as a chronic stressor that triggers psychological responses [42]. The current finding may indicate that public stigma may not only exert direct impact on psychological well-being but may also create a cascade of stress responses that amplify its effects.

Moderation analysis revealed that the link from public stigma to perceived stress was significantly stronger among individuals with no formal education and those who sought help from mental health professionals and herbalists. Furthermore, the findings highlight educational attainment as a protective factor. Education can enhance problem-solving skills and coping mechanisms that buffer the psychological effects of stigma [44]. These findings also highlight the vulnerability of individuals with no or limited formal education to the effects of public stigma, possibly due to limited resources to manage stigma-related stress [44]. With regards to participants who sought help for mental health services, the findings may reflect the heightened need to seek help either from herbalists/spiritualists or health professionals due to the severe impact of public stigma.

The findings of the study have several implications for skin-NTDs care and psychosocial interventions. The study indicated a high burden of psychological distress, perceived stress and public stigma in individuals living with skin-NTDs. Our structural equation model further reveals that public stigma is not only prevalent but also contributes significantly to perceived stress and psychological distress, highlighting the potential for psychosocial harm to perpetuate or worsen disease outcomes. Given these findings, there is a strong reason to integrate mental health care and support into primary services and skin-NTDs control and elimination programmes. Public stigma and psychological distress can delay seeking medical help, contribute to non-adherence, and threaten contact tracing [45,46]. Therefore, interventions that reduce public stigma and address psychosocial needs of individuals living with skin-NTDS may enhance early detection, community participation and treatment success rates, ultimately contributing to effective skin-NTDS control and/or elimination. We also recommend training frontline skin-NTD healthcare providers to identify and refer individuals for mental health support. Skin-NTD outreach programs could also be embedded into the training modules of community mental health support volunteers for improved mental health services coverage, especially in community settings. Our findings also suggest that routine screening for psychological distress, perceived stress and public stigma experiences as part of primary and community healthcare services would help in early identification of at-risk individuals for early intervention. This brings to light the need to validate and establish the psychometric properties of screening measures for use among skin-NTD populations. The proposal for integrated healthcare, comprising mental and physical health, aligns directly with the WHO Roadmap on Neglected Tropical Diseases 2021–2030, WHO Mental Health Action Plan 2013–2030, WHO Mental Health Gap Action Programme and WHO Quality Rights initiative (WHO, 2020) and the Mental Health Policy 2019–2030 of the Ministry of Health-Ghana.

### Limitations and recommendations for future studies

The cross-sectional design limits the ability to establish causality and the cascading effects that emerged. The study also relied on self-reported data that may be subject to recall bias and social desirability, thereby limiting the validity of the estimated burden of psychological distress and perceived stress among individuals with skin- NTDs. The small sample sizes for subgroups could potentially reduce the statistical power of the moderation analysis, thereby affecting the statistical significance of the results.

Closely related to the above is the data collection measures which have been validated among different populations in Africa and outside Africa, with none of the validation studies conducted among individuals with skin-NTDs in Ghana. This observation raises the possibility that the participants might under/over-report the behaviours indexed by the data collection measures.

Beyond addressing the limitations reported above, future longitudinal studies are needed to confirm the temporal relationships of observed pathways (e.g., Stigma precedes stress and psychological distress). Our study revealed that participants who reported high psychological distress were more likely to seek mental health support, compared with their counterparts who score low on psychological distress. This observation could result from several factors, including the likelihood that the high psychological distress motivated help-seeking behaviours, or such individuals are internally motivated to consume mental health services, or both. Qualitative and longitudinal observational studies could further enrich our understanding of why skin-NTD patients who seek mental health have a higher burden of perceived stress and psychological distress compared to their counterparts who do not seek such help.

## Conclusion

While ongoing initiatives to eradicate skin-NTDs are highly commendable and recommended, it is equally important to pay attention to the psychosocial issues occasioned by the diagnoses of skin-NTDs. The burden of psychological distress and perceived stress among individuals affected by skin-NTDs is reportedly high, an observation that is consistent with existing literature. The finding also presents important interactions and moderating factors that influence the relationship between psychological distress, perceived stress and public stigma. Understanding the nature and mechanisms of psychological distress among individuals affected by skin-NTDs to inform efforts to improve the psychosocial wellbeing and quality of this vulnerable population may contribute to the overall goal of eradicating skin-NTDs in Ghana and other jurisdictions. Consequently, we conclude by inviting additional studies to focus on the psychological distress and other wellbeing issues faced by skin-NTD population. The findings further highlight the need for integrated, culturally sensitive interventions that address psychological distress and public stigma experiences among individuals affected by skin-NTDs.
